# Urban–rural disparities in hypertension prevalence, awareness, treatment, and control among Chinese middle-aged and older adults from 2011 to 2015: a repeated cross-sectional study

**DOI:** 10.1186/s12872-022-02769-5

**Published:** 2022-07-17

**Authors:** Haozhe Cheng, Yiran Gu, Xiaochen Ma, Haoqing Tang, Xiaoyun Liu

**Affiliations:** 1 School of Public Health, Peking University, Beijing, 100191 China; 2 Division of Environmental Health Science, School of Public Health, University of California, Berkeley, Berkeley, CA 94720 USA; 3 China Center for Health Development Studies, Peking University, Beijing, 100191 China

**Keywords:** Urban–rural disparities, Hypertension, China, Middle-aged and older adults

## Abstract

**Background:**

China has experienced a continuing increase in hypertension prevalence over the past few decades, especially in rural areas. The paper aims to examine the variation of urban–rural disparities in hypertension prevalence, awareness, treatment, and control among Chinese middle-aged and older adults between 2011 and 2015.

**Methods:**

Our team extracted data from the China Health and Retirement Longitudinal Study (CHARLS), a nationally representative survey of residents aged 45 years and older. In this study, we used the 2011 wave and the 2015 wave of CHARLS. We calculated crude rates and age-adjusted rates of hypertension prevalence, awareness, treatment, and control for the general, urban, and rural populations in each wave and performed chi-square tests to examine urban–rural disparities. We used logistic regression to further confirm these disparities by controlling confounding factors in each wave. We then used generalized estimating equation (GEE) to further examine whether urban–rural disparities changed between 2011 and 2015.

**Results:**

We included 11,129 records in the 2011 wave and 8916 records in the 2015 wave in this study. The mean age was 59.0 years and 5359 (48.2%) participants were male in the 2011 wave. Age-adjusted hypertension prevalence, awareness, treatment, control, and control among treated in the total population were 38.5%, 70.6%, 59.2%, 27.4%, and 46.4% in 2015. Urban–rural disparities in the indicators mentioned above were 5.7%, 13.4%, 15.3%, 9.4% and 5.6% in 2011; which decreased to 4.8%, 2.7%, 5.2%, 4.9% and 3.8% in 2015. Urban–rural disparities in prevalence, awareness and treatment were statistically significant in 2011 but not significant in 2015 adjusted for confounding factors, yet control disparities were statistically significant in both waves. Finally, urban–rural disparities in awareness and treatment had narrowed from 2011 to 2015.

**Conclusions:**

Awareness, treatment, and control rates were sub-optimal among both urban and rural adults. Prevention and management of hypertension among both urban and rural adults should be further strengthened. Awareness and treatment increased more rapidly among rural adults, indicating some achievement had been made in enhancing the healthcare system in rural areas. More efforts are needed in attaining urban–rural equity of healthcare services.

## Background

Hypertension, a leading risk factor for cardiovascular disease (CVD), is an important cause of disability and mortality worldwide [1, 2]. 31.1% of adults (1.39 billion) worldwide had hypertension in 2010 [3]. As the biggest developing country contributing to approximately 20% of the world’s population, China is experiencing a heavy disease burden of hypertension, which accounted for 14.28% of disability-adjusted life-years (DALYs) and 27.5% of deaths in 2013 [4]. The prevalence of hypertension in China has been increasing rapidly in the past few decades [5–7]. Notably, the rates of hypertension awareness, treatment, and control, important indicators that mirror the management abilities of the health care system, were still very low in China [8–11].

China has experienced rapid development since the market economy reform in 1978, which led to the transition of the traditional lifestyle, the increasing burden of non-communicable diseases was firstly concentrated in cities and then gradually spread to rural towns and villages [12]. With the rapid urbanization of rural areas, sedentary behaviors [13] and intake of high calorific value food [14] of rural residents have been continuing to increase. Hypertension prevalence in rural adults increased rapidly during the past few decades [15] and caught up with that of urban adults recently [8]. However, hypertension awareness, treatment, and control were always found to be lower among rural adults in China [8, 9, 16], which might mainly be due to the weak health system, poor healthcare human resources, and insufficient government investment in rural areas [17]. In 2009, a thorough reform of the national health care system was initiated to better manage non-communicable diseases (NCDs) and promote health equity [18]. For example, establishing a basic national public health service program (BNPHSP) was an important measure of this reform [10], which has made a great change to the prevention and management of non-communicable diseases in rural areas [19].

The development trend and urban–rural disparities of hypertension prevalence, awareness, treatment, and control among urban and rural adults after 2011 were not clear to date. This paper aims to compare the urban–rural differences in hypertension prevalence, awareness treatment, and control in 2011 and 2015, and whether their urban–rural difference has changed from 2011 to 2015 after the 2009 health system reform, to mirror the variation of urban–rural inequity in health care and draw public policy implications for further reform of health care systems.

## Method

### Data source

Data for this study was extracted from 2 waves (2011 and 2015) of the China Health and Retirement Longitudinal Study (CHARLS), an ongoing nationwide survey of residents aged 45 years and older from 28 provinces in China [20]. CHARLS was first collected in 2011 and then followed up every 2–3 years. Multistage stratified probability-proportionate-to-size (PPS) sampling was applied to generate a nationally representative sample in baseline, and a supplement sample was added in each follow-up wave to maintain the national representativeness of Chinese adults aged 45 years and older. A well-structured questionnaire was used and body examination was taken to collect demographic and health information in both waves. Further details of the CHARLS have been described elsewhere [20].

### Participants

The original sample included 17,708 records in the 2011 wave and 21,097 adults in the 2015 wave. The inclusion criteria are as follows: (1) people who are not less than 45 years old; (2) people who reported whether had hypertension diagnosed by a doctor and reported whether being taking anti-hypertensive medication; (3) people who measured blood pressure 3 times; (4) people with no missing data of covariates.

### Variables

The main outcome variables of this study were hypertension prevalence, awareness, treatment, and control. The systolic blood pressure (SBP) and diastolic blood pressure (DBP) of each respondent were recorded 3 times in the sitting position by a trained nurse using an HEM-7112 electronic monitor (Omron, Kyoto, Japan). The mean of the 3 readings was recorded as their BP values. Hypertension was defined as mean SBP ≥ 140 mmHg and/or mean DBP ≥ 90 mmHg and/or self-report taking anti-hypertensive medication currently. Hypertensive respondents who answered “yes” to the question “have you been diagnosed with hypertension by a doctor?” were defined as aware of hypertension. Hypertensive respondents who claimed currently taking antihypertensive medication were defined as treated. Hypertensive respondents who had a mean SBP of < 140 mmHg and a mean DBP of < 90 mmHg were defined as controlled [10, 21].

This study’s major independent variable of interest is urban/rural, which indicates whether a respondent belongs to urban or rural registration. A specific national administrative household registration system called “hukou” was originally designed to prohibit population migration in 1958 in China [22]. Hundreds of millions of rural workers have migrated to urban areas since the Chinese market-oriented reforms launched in the 1990s. By 2017, there were 286.5 million rural-to-urban migrant workers in China [23], which contribute to about one-fifth of China’s total population, but very few of them could get an urban hukou [22]. Residents holding an urban hukou not only have better chances in the labor market, but also can get numerous social security benefits such as housing subsidies, retirement allowances, unemployment insurance, and health insurance [10, 15, 22].

We controlled demographic and socioeconomic variables of this study including sex, age (categorized into 45–54, 55–64, 65–74, and ≥ 75), region (categorized into eastern, central, and western), marital status (categorized into married/partnered, and not partnered), education (categorized into illiterate, primary school and below, and secondary school and above), and household consumption per capita (the whole sample were ranked by household consumption per capita and were divided into 5 quintiles in each year to indicate relative economic level) [8, 15]. We also controlled some risk factors of hypertension such as smoke (classified into never, quit, current), drink (classified into never, quit, current), and body-mass-index(BMI) group (classified into < 24, 24–28, ≥ 28 kg/m^2^ according to Chinese standard [24], [25, 26].

### Statistical analysis

We carried out descriptive statistics and multivariate regression in this study. Data analyses were conducted using STATA 16.0 and R4.1.1.

Descriptive statistics for all covariates of total, urban and rural adults were presented as proportions in each year. We reported numbers of adults, crude rate, and age-adjusted rate of hypertension prevalence, awareness, treatment control, and control among treated hypertensive adults by total, urban and rural. We used the chi-square test was used to examine whether there were statistical differences between urban and rural adults, with *p*-values presented.

Logistic regression in each wave was adopted to investigate whether urban–rural disparities of prevalence, awareness, treatment, control, and control among treated hypertensive adults existed in each wave after controlling for covariates. The model was specified as: *ln *$(\frac{{P_{i} }}{{1 - P_{i} }})$ = *β*_*0*_ + *β*_*1*_*rural* + *β*_*i*_*X*_*i*_ + *u*. *P*_*i*_ was the probability of hypertension prevalence, awareness, treatment, or control; *rural* was the dummy for hukou type; *X*_*i*_ were covariates. Odds ratios were reported.

To explore whether urban–rural disparities of prevalence, awareness, treatment, control, and control among treated hypertensive adults had changed between 2011 and 2015, we adopted generalized estimating equation (GEE) regression for longitudinal data[27], of which the link function was set as “logit”. Differences in urban–rural disparities changed between 2011 and 2015 were examined by adding an interaction term of urban/rural and wave dummy variable. The model was specified as: *ln *$(\frac{{P_{i} }}{{1 - P_{i} }})$ = *β*_*0*_ + *β*_*1*_*rural* + *β*_*2*_*2015wave* + *δ*_*1*_rural*2015wave + *β*_*i*_*X*_*i*_ + *u*. *P*_*i*_ was the probability of hypertension prevalence, awareness, treatment, or control; *rural* was the dummy variable for urban/rural; *wave* was the dummy variable for wave; rural*2015wave was an interaction term of urban/rural and wave dummy variable; *X*_*i*_ were covariates. *β*_*1*_ captured the effect of rural hukou on outcome indicators in the 2011 wave; *β*_*2*_ captured the change of outcome indicators from 2011 to 2015 in urban adults*; δ*_*1*_ captured the change of urban–rural disparity of outcome indicators between the 2 waves; (*β*_*1*_ + *δ*_*1*_) captured the effect of rural hukou on outcome indicators in the 2015 wave. Odds ratios were reported.

## Results

### Descriptive study

The final sample included in this study were 11,129 records in the 2011 wave and 8916 records in the 2015 wave. The distribution of demographic characteristics is displayed in Table 1. In the 2011 wave, the mean age of all participants was 59.0 years, 5359 (48.2%) participants were male, and 5770 (51.8%) participants were female. Urban adults contributed to approximately 20% of the whole sample (2103/11,129 in the 2011 wave, 1591/8916 in the 2015 wave). Socioeconomic status is distributed differently among urban and rural adults. For example, the highest education level group and economic level group contributed to 60.7% and 40.8% of the whole sample among urban adults, while only 24.5% and 15.0% of the whole sample among rural adults in the 2011 wave.

**Table 1:** Demographic Characteristics of study population [n(%)]

Variables	2011 wave			2015 wave		
	Total (n = 11,129)	Urban (n = 2103)	Rural (n = 9026)	Total (n = 8916)	Urban (n = 1591)	Rural (n = 7325)
Sex						
Male	5359 (48.2%)	1107 (52.6%)	4252 (47.1%)	4200 (47.1%)	844 (53.0%)	3356 (45.8%)
Female	5770 (51.8%)	996 (47.4%)	4774 (52.9%)	4716 (52.9%)	747 (47.0%)	3969 (54.2%)
Age group						
45–54	3953 (35.52%)	654 (31.10%)	3299 (36.55%)	2181 (24.46%)	354 (22.25%)	1827 (24.94%)
55–64	4253 (38.22%)	799 (37.99%)	3454 (38.27%)	3504 (39.30%)	595 (37.40%)	2909 (39.71%)
65–74	2100 (18.87%)	456 (21.68%)	1644 (18.21%)	2328 (26.11%)	453 (28.47%)	1875 (25.60%)
75 +	823 (7.40%)	194 (9.22%)	629 (6.97%)	903 (10.13%)	189 (11.88%)	714 (9.75%)
Region						
Eastern	3626 (32.6%)	633 (30.1%)	2993 (33.2%)	2965 (33.3%)	452 (28.4%)	2513 (34.3%)
Central	3886 (34.9%)	873 (41.5%)	3013 (33.4%)	3008 (33.7%)	645 (40.5%)	2363 (32.3%)
Western	3617 (32.5%)	597 (28.4%)	3020 (33.5%)	2943 (33.0%)	494 (31.0%)	2449 (33.4%)
Marital status						
Married	9748 (87.6%)	1844 (87.7%)	7904 (87.6%)	7716 (86.5%)	1399 (87.9%)	6317 (86.2%)
Not partnered	1381 (12.4%)	259 (12.3%)	1122 (12.4%)	1200 (13.5%)	192 (12.1%)	1008 (13.8%)
Education						
Illiterate	3117 (28.0%)	210 (10.0%)	2907 (32.2%)	2442 (27.4%)	149 (9.4%)	2293 (31.3%)
Primary and below	4522 (40.6%)	616 (29.3%)	3906 (43.3%)	3672 (41.2%)	479 (30.1%)	3193 (43.6%)
Secondary and above	3490 (31.4%)	1277 (60.7%)	2213 (24.5%)	2802 (31.4%)	963 (60.5%)	1839 (25.1%)
Household consumption per capita						
Quintile 1 (poorest)	2217 (19.9%)	147 (7.0%)	2070 (22.9%)	1748 (19.6%)	138 (8.7%)	1610 (22.0%)
Quintile 2	2231 (20.0%)	234 (11.1%)	1997 (22.1%)	1794 (20.1%)	181 (11.4%)	1613 (22.0%)
Quintile 3	2225 (20.0%)	334 (15.9%)	1891 (21.0%)	1794 (20.1%)	247 (15.5%)	1547 (21.1%)
Quintile 4	2241 (20.1%)	531 (25.2%)	1710 (18.9%)	1778 (19.9%)	371 (23.3%)	1407 (19.2%)
Quintile 5 (richest)	2215 (19.9%)	857 (40.8%)	1358 (15.0%)	1802 (20.2%)	654 (41.1%)	1148 (15.7%)
Smoke status						
Never	6642 (59.7%)	1281 (60.9%)	5361 (59.4%)	4956 (55.6%)	868 (54.6%)	4088 (55.8%)
Quit	1047 (9.4%)	243 (11.6%)	804 (8.9%)	1492 (16.7%)	305 (19.2%)	1187 (16.2%)
Current	3440 (30.9%)	579 (27.5%)	2861 (31.7%)	2468 (27.7%)	418 (26.3%)	2050 (28.0%)
Drink status						
Never	6528 (58.7%)	1220 (58.0%)	5308 (58.8%)	4878 (54.7%)	807 (50.7%)	4071 (55.6%)
Quit	933 (8.4%)	203 (9.7%)	730 (8.1%)	1029 (11.5%)	189 (11.9%)	840 (11.5%)
Current	3668 (33.0%)	680 (32.3%)	2988 (33.1%)	3009 (33.7%)	595 (37.4%)	2414 (33.0%)
Body mass index groups						
< 24	6652 (59.8%)	1005 (47.8%)	5647 (62.6%)	4920 (55.2%)	725 (45.6%)	4195 (57.3%)
24–28	3234 (29.1%)	785 (37.3%)	2449 (27.1%)	2881 (32.3%)	618 (38.8%)	2263 (30.9%)
> = 28	1243 (11.2%)	313 (14.9%)	930 (10.3%)	1115 (12.5%)	248 (15.6%)	867 (11.8%)

Table 2 reports hypertension prevalence, awareness, treatment, and control grouped by urban/rural and waves. Age-adjusted hypertension prevalence, awareness, treatment, control, and control among treated in the total population were 38.5%, 70.6%, 59.2%, 27.4%, and 46.4% in 2015. The rate of awareness, treatment, control, and control among treated patients increased from 2011 to 2015 among both urban and rural adults. Urban–rural gaps of age standard rates of prevalence, awareness, treatment, control and control among treated patients were 5.7%, 13.4%, 15.3%, 9.4% and 5.6% in 2011; and had decreased to 4.8%, 2.7%, 5.2%, 4.9%, and 3.8% in 2015. Chi-square tests showed that prevalence, awareness, treatment control, and control among treated patients with hypertension were higher among urban adults than rural adults in both waves (p < 0.05).

**Table 2:** Prevalence, awareness, treatment, and control among the study population

	2011 wave				2015 wave			
	Total	Urban	Rural	*P* value	Total	Urban	Rural	*P* value
Prevalence								
n/N	4299 / 11,129	942 / 2103	3357 / 9026	< 0.001	3689 / 8916	723 / 1591	2966 / 7325	< 0.001
Crude rate (95% CI)	38.6 (37.7–39.5)	44.8 (42.7–46.9)	37.2 (36.2–38.2)		41.4 (40.4–42.4)	45.4 (43.0–47.9)	40.5 (39.4–41.6)	
Age-adjusted rate (95% CI)	38.2 (37.3–39.1)	42.8 (40.7–44.8)	37.1 (36.1–38.1)		38.5 (37.5–39.6)	42.5 (39.9–45.2)	37.7 (36.5–38.8)	
Awareness								
n/N	2497 / 4299	644 / 942	1853 / 3357	< 0.001	2648 / 3689	546 / 723	2102 / 2966	0.013
Crude rate (95% CI)	58.1 (56.6–59.6)	68.4 (65.4–71.3)	55.2 (53.5–56.9)		71.8 (70.3–73.2)	75.5 (72.4–78.7)	70.9 (69.2–72.5)	
Age-adjusted rate (95% CI)	57.2 (55.6–58.8)	67.8 (64.4–71.2)	54.4 (52.6–56.2)		70.6 (68.9–72.4)	72.8 (68.8–76.8)	70.1 (68.1–72.1)	
Treatment								
n/N	2123 / 4299	584 / 942	1539 / 3357	< 0.001	2251 / 3689	480 / 723	1771 / 2966	< 0.001
Crude rate (95% CI)	49.4 (47.9–50.9)	62.0 (58.9–65.1)	66.4 (62.9–69.8)		61.0 (59.4–62.6)	45.8 (44.2–47.5)	59.7 (57.9–61.5)	
Age-adjusted rate (95% CI)	48.2 (46.6–49.8)	60.2 (56.7–63.8)	44.9 (43.1–46.7)		59.2 (57.2–61.1)	63.3 (59.0–67.6)	58.1 (56.0–60.2)	
Control								
n/N	889 / 4299	259 / 942	630 / 3357	< 0.001	1004 / 3689	233 / 723	771 / 2966	< 0.001
Crude rate (95% CI)	20.7 (19.5–21.9)	27.5 (24.6–30.3)	18.8 (17.4–20.1)		27.2 (25.8–28.7)	32.2 (28.8–35.6)	26.0 (24.4–27.6)	
Age-adjusted rate (95% CI)	20.4 (19.1–21.6)	27.8 (24.6–31.0)	18.4 (17.0–19.8)		27.4 (25.6–29.1)	31.3 (27.3–35.3)	26.4 (24.5–28.4)	
Control among treated								
n/N	889 / 2123	259 / 584	630 / 1539	< 0.001	1004 / 2251	233 / 480	771 / 1771	< 0.001
Crude rate (95% CI)	41.9 (39.8–44.0)	44.3 (40.3–48.4)	40.9 (38.5–43.4)		44.6 (42.5–46.7)	48.5 (44.1–53.0)	43.5 (41.2–45.8)	
Age-adjusted rate (95% CI)	42.1 (39.8–44.4)	46.3 (41.6–51.0)	40.7 (38.1–43.4)		46.4 (43.9–49.0)	49.5 (43.8–55.2)	45.7 (42.8–48.5)	

### Logistic regression analysis

The effects of the hukou dummy variable in Tables 3 and 4 present the logistic regression estimates of urban–rural disparities of hypertension prevalence, awareness, treatment, and control adjusted for all covariates in each wave. Rural adults were less likely to be hypertensive in both waves, but this trend is only statistically significant in the 2011 wave (OR 0.817, 95% CI 0.729–0.916) while not statistically significant in the 2015 wave (OR 0.933, 95% CI 0.822–1.060). The probabilities of awareness, treatment, and control among rural hypertensive adults were also lower than urban counterparts in both waves, but the disparities of awareness (OR 0.932, 95% CI 0.754–1.149) and treatment (OR 0.844, 95% CI 0.694–1.024) in 2015 wave were not statistically significant. The difference of control among hypertensive adults in treatment was not evident between urban and rural adults.

**Table 3:** Logistic regression estimates of hypertension prevalence and awareness [Odds Ratio(95%CI)]

	Prevalence		Awareness	
	2011 wave	2015 wave	2011 wave	2015 wave
Hukou (ref: Urban)				
Rural	0.817 (0.729–0.916)*	0.933 (0.822–1.060)	0.750 (0.630–0.892)*	0.932 (0.754–1.149)
Sex (ref: Male)				
Female	1.132 (1.002–1.280)*	1.025 (0.887–1.184)	1.246 (1.033–1.503)*	1.182 (0.925–1.509)
Age (ref: 45–54)				
55–64	1.906 (1.722–2.112)*	1.709 (1.512–1.934)*	1.505 (1.270–1.784)*	1.229 (0.986–1.529)
65–74	3.172 (2.800–3.594)*	3.007 (2.620–3.454)*	1.539 (1.268–1.871)*	1.736 (1.366–2.206)*
75+	4.688 (3.919–5.616)*	4.190 (3.471–5.064)*	1.375 (1.066–1.774)*	1.370 (1.014–1.852)*
Region (ref: Eastern)				
Central	0.853(0.773–0.942)*	0.911(0.817–1.016)	0.982(0.842–1.145)	0.978(0.818–1.169)
Western	0.903(0.817–0.999)*	0.913(0.817–1.019)	0.808(0.692–0.945)*	1.114(0.926–1.340)
Marital status (ref: Married)				
Not partnered	1.393(1.227–1.581)*	1.286(1.121–1.474)*	0.874(0.730–1.047)	0.978(0.790–1.214)
Education (ref: Illiterate)				
Primary	0.958(0.862–1.065)	0.967(0.861–1.086)	1.038(0.883–1.220)	1.056(0.869–1.281)
Secondary and above	0.981(0.864–1.115)	0.954(0.830–1.097)	1.213(0.992–1.485)	1.113(0.877–1.413)
Household consumption per capita [ref: Quintile 1 (poorest)]				
Quintile 2	0.874(0.770–0.993)*	0.933(0.810–1.074)	0.920(0.759–1.115)	0.935(0.745–1.173)
Quintile 3	0.878(0.773–0.998)*	1.029(0.894–1.185)	1.159(0.954–1.408)	1.170(0.931–1.471)
Quintile 4	0.872(0.766–0.993)*	0.992(0.860–1.145)	1.216(0.998–1.481)	1.205(0.954–1.524)
Quintile 5 (richest)	0.751(0.656–0.860)*	0.965(0.833–1.119)	1.527(1.236–1.889)*	1.518(1.185–1.948)*
Smoke status (ref: Never)				
Quit	1.078(0.919–1.264)	1.237(1.059–1.445)*	1.127(0.889–1.432)	1.058(0.820–1.367)
Current	1.080(0.958–1.217)	1.125(0.974–1.299)	0.897(0.747–1.077)	0.941(0.740–1.197)
Drink status (ref: Never)				
Quit	1.433(1.229–1.670)*	1.159(0.997–1.348)	1.660(1.316–2.102)*	2.025(1.542–2.687)*
Current	1.079(0.972–1.198)	1.066(0.953–1.193)	0.933(0.789–1.104)	0.777(0.644–0.939)*
Body mass index groups (ref: < 24)				
24–28	2.101(1.913–2.308)*	2.317(2.094–2.564)*	1.596(1.381–1.846)*	1.721(1.456–2.036)*
> = 28	4.440(3.883–5.082)*	4.211(3.648–4.866)*	2.462(2.035–2.988)*	2.533(2.012–3.207)*

*P value < 0.05

**Table 4:** Logistic regression estimates of hypertension treatment and control [Odds Ratio(95%CI)]

	Treatment		Control		Control among treated	
	2011 wave	2015 wave	2011 wave	2015 wave	2011 wave	2015 wave
Hukou (ref: Urban)						
Rural	0.689(0.581–0.816)*	0.844(0.694–1.024)	0.752(0.620–0.914)*	0.805(0.660–0.983)*	0.894(0.714–1.119)	0.844(0.671–1.061)
Sex (ref: Male)						
Female	1.165(0.966–1.404)	1.585(1.264–1.991)*	1.084(0.868–1.357)	1.426(1.118–1.823)*	0.990(0.761–1.287)	1.170(0.889–1.542)
Age (ref: 45–54)						
55–64	1.657(1.398–1.966)*	1.513(1.231–1.860)*	1.490(1.220–1.825)*	1.116(0.895–1.394)	1.132(0.892–1.437)	0.869(0.67–1.127)
65–74	1.694(1.394–2.060)*	1.924(1.541–2.405)*	1.147(0.905–1.453)	1.132(0.895–1.435)	0.787(0.597–1.038)	0.760(0.577–1.001)
75+	1.541(1.192–1.993)*	1.512(1.141–2.006)*	0.696(0.490–0.980)*	0.913(0.667–1.247)	0.445(0.300–0.654)*	0.664(0.465–0.948)*
Region (ref: Eastern)						
Central	0.884(0.759–1.029)	0.957(0.810–1.131)	0.871(0.729–1.040)	0.957(0.799–1.145)	0.911(0.741–1.119)	0.962(0.783–1.181)
Western	0.705(0.603–0.825)*	0.969(0.817–1.151)	0.650(0.535–0.788)*	1.031(0.858–1.238)	0.748(0.597–0.936)	1.064(0.862–1.313)
Marital status (ref: Married)						
Not partnered	0.954(0.795–1.144)	0.860(0.705–1.050)	0.899(0.710–1.131)	0.756(0.603–0.945)*	0.906(0.693–1.183)	0.765(0.595–0.980)*
Education (ref: Illiterate)						
Primary	1.119(0.952–1.316)	1.178(0.984–1.411)	1.150(0.941–1.406)	1.304(1.074–1.585)*	1.091(0.864–1.377)	1.266(1.019–1.574)*
Secondary and above	1.345(1.101–1.644)*	1.270(1.017–1.586)*	1.309(1.032–1.662)*	1.266(0.999–1.606)	1.131(0.857–1.495)	1.161(0.890–1.515)
Household consumption per capita [ref: Quintile 1 (poorest)]						
Quintile 2	1.018(0.838–1.238)	1.021(0.823–1.267)	1.104(0.858–1.422)	0.957(0.749–1.223)	1.157(0.863–1.554)	0.940(0.712–1.241)
Quintile 3	1.126(0.926–1.370)	1.059(0.855–1.312)	1.237(0.965–1.587)	1.111(0.876–1.409)	1.251(0.936–1.672)	1.109(0.846–1.454)
Quintile 4	1.163(0.954–1.417)	1.177(0.945–1.466)	1.338(1.046–1.713)*	1.304(1.028–1.655)*	1.342(1.007–1.789)	1.278(0.974–1.678)
Quintile 5 (richest)	1.433(1.162–1.768)*	1.335(1.063–1.678)*	1.474(1.142–1.905)*	1.290(1.011–1.647)*	1.296(0.965–1.741)	1.174(0.889–1.552)
Smoke status (ref: Never)						
Quit	1.189(0.941–1.505)	1.209(0.956–1.532)	1.183(0.901–1.548)	1.249(0.973–1.603)	1.110(0.812–1.516)	1.194(0.899–1.586)
Current	0.824(0.686–0.991)	1.057(0.845–1.323)	0.788(0.628–0.988)*	1.101(0.861–1.407)	0.864(0.662–1.127)	1.137(0.861–1.502)
Drink status (ref: Never)						
Quit	1.519(1.212–1.907)*	2.154(1.690–2.761)*	1.374(1.060–1.774)*	1.513(1.201–1.903)*	1.090(0.810–1.466)	1.088(0.842–1.406)
Current	0.831(0.701–0.984)*	0.750(0.629–0.895)*	0.901(0.731–1.109)	0.786(0.644–0.958)*	1.003(0.783–1.283)	0.903(0.716–1.138)
Body mass index groups (ref: < 24)						
24–28	1.667(1.443–1.927)*	1.583(1.355–1.850)*	1.204(1.009–1.436)*	1.133(0.956–1.343)	0.825(0.669–1.015)	0.860(0.708–1.046)
> = 28	2.871(2.383–3.465)*	2.495(2.024–3.085)*	1.380(1.117–1.702)*	1.336(1.083–1.645)*	0.679(0.533–0.863)*	0.819(0.648–1.035)

*P value < 0.05

### Generalized estimating equation (GEE) analysis

Variations of urban–rural disparities in hypertension prevalence, awareness, treatment, and control from 2011 to 2015 were represented by the interaction term of urban/rural and wave in GEE analysis and were shown in Table 5. It can be discovered that the urban–rural disparities of hypertension awareness (OR 1.291, 95% CI 1.010–1.651) and treatment (OR 1.330, 95% CI 1.053–1.680) had significantly narrowed.

**Table 5:** GEE regression estimates of hypertension prevalence, awareness, treatment, and control [Odds Ratio(95%CI)]

	Prevalence	Awareness	Treatment	Control	Control among treated
Hukou (ref: urban)					
Rural	0.842(0.756–0.939)*	0.738(0.624–0.874)*	0.666(0.565–0.785)*	0.729(0.608–0.874)*	0.891(0.724–1.096)
Wave (ref: 2011 wave)					
2015 wave	0.882(0.767–1.015)	1.410(1.129–1.760)*	1.200(0.973–1.480)	1.279(1.031–1.586)*	1.216(0.952–1.553)
Hukou * Wave					
Rural * 2015wave	1.070(0.917–1.249)	1.291(1.010–1.651)*	1.330(1.053–1.680)*	1.134(0.887–1.451)	0.939(0.709–1.243)
Sex (ref: Male)					
Female	1.081(0.985–1.185)	1.221(1.053–1.416)*	1.319(1.142–1.525)*	1.225(1.037–1.447)*	1.071(0.886–1.296)
Age (ref: 45–54)					
55–64	1.829(1.691–1.977)*	1.372(1.199–1.569)*	1.571(1.378–1.791)*	1.284(1.107–1.490)*	0.993(0.834–1.182)
65–74	3.131(2.857–3.432)*	1.623(1.396–1.887)*	1.787(1.543–2.070)*	1.140(0.966–1.345)	0.776(0.640–0.942)*
75 +	4.465(3.922–5.082)*	1.378(1.136–1.670)*	1.522(1.260–1.838)*	0.828(0.658–1.041)	0.571(0.441–0.740)*
Region (ref: Eastern)					
Central	0.881(0.818–0.948)*	0.979(0.871–1.100)	0.913(0.816–1.023)	0.914(0.805–1.037)	0.939(0.813–1.086)
Western	0.908(0.843–0.978)*	0.927(0.823–1.044)	0.815(0.727–0.914)*	0.830(0.727–0.947)*	0.910(0.781–1.059)
Marital status (ref: Married)					
Not partnered	1.338(1.218–1.478)*	0.918(0.801–1.052)	0.916(0.801–1.047)	0.827(0.704–0.971)*	0.823(0.686–0.987)*
Education (ref: Illiterate)					
Primary and below	0.963(0.891–1.041)	1.054(0.932–1.192)	1.153(1.023–1.300)*	1.239(1.077–1.425)*	1.188(1.014–1.392)
Secondary and above	0.97(0.882–1.065)	1.162(0.997–1.355)	1.303(1.123–1.511)*	1.281(1.079–1.519)*	1.140(0.940–1.382)
Household consumption per capita [ref: Quintile 1 (poorest)]					
Quintile 2	0.901(0.819–0.991)*	0.934(0.807–1.081)	1.029(0.891–1.189)	1.034(0.867–1.233)	1.034(0.845–1.265)
Quintile 3	0.943(0.858–1.037)	1.166(1.006–1.351)*	1.094(0.948–1.263)	1.169(0.985–1.388)	1.165(0.957–1.419)
Quintile 4	0.924(0.839–1.018)	1.212(1.042–1.409)*	1.172(1.012–1.356)*	1.320(1.113–1.566)*	1.300(1.068–1.581)*
Quintile 5 (richest)	0.842(0.762–0.931)*	1.521(1.294–1.788)*	1.395(1.195–1.629)*	1.379(1.156–1.645)*	1.217(0.995–1.489)
Smoke status (ref: Never)					
Quit	1.169(1.048–1.305)*	1.091(0.920–1.293)	1.156(0.980–1.364)	1.182(0.987–1.415)	1.141(0.928–1.403)
Current	1.099(1.003–1.204)*	0.917(0.792–1.060)	0.908(0.787–1.046)	0.917(0.777–1.083)	0.989(0.817–1.197)
Drink status (ref: Never)					
Quit	1.280(1.149–1.426)*	1.827(1.534–2.176)*	1.805(1.531–2.128)*	1.467(1.237–1.740)*	1.101(0.908–1.336)
Current	1.070(0.992–1.154)	0.866(0.763–0.983)*	0.794(0.703–0.898)*	0.840(0.727–0.972)*	0.952(0.803–1.129)
Body mass index groups (ref: < 24)					
24–28	2.196 (2.050–2.352)*	1.654 (1.483–1.845)*	1.630 (1.467–1.812)*	1.169 (1.035–1.322)*	0.847 (0.735–0.976)*
> = 28	4.325 (3.917–4.774)*	2.506 (2.161–2.906)*	2.710 (2.358–3.115)*	1.368 (1.180–1.586)*	0.751 (0.635–0.888)*

*P value < 0.05

## Discussion

Overall, this study found that rural adults had lower hypertension prevalence, awareness, treatment, and control in both waves. However, urban–rural gaps in hypertension awareness and treatment had evidently narrowed from 2011 to 2015, and urban–rural disparities in prevalence, awareness, and treatment were not statistically significant after controlling for confounding factors in the 2015 wave.

This study found that the prevalence of hypertension was higher among urban adults in both waves among Chinese adults aged 45 years old and above, a conclusion similar to most studies of low-income countries [28–31] but opposite to that of high-income countries [28]. In China, hypertension prevalence was lower among rural adults than urban counterparts between 1990 and 2010 [5, 15], while some recently published studies found that the prevalence of rural adults was almost the same as [9] or even higher than that of urban adults [8, 32–34]. A possible explanation is these studies were either based on adults of all ages or not nationally representative, but the rapid increase of hypertension prevalence among rural adults always needs to pay attention to [15].

This study also found that the rates of awareness, treatment, and control were higher among urban adults, which is in accordance with most existing studies of China [15, 33, 35] and some low-income and middle-income countries [28, 36, 37]. With the unbalanced development between urban and rural areas since 1978, the gap in the healthcare system between urban and rural areas has widened [38], as well as the health care human resources. In 2011, there were 7.10 and 3.19 health professionals per 1000 population in urban areas and rural areas, respectively [39]. Some rural residents had to cover a long distance to seek for medical service. Besides, one’s urban/rural registration largely determined the type of medical insurance to be covered. Rural residents were mostly covered by New Cooperative Medical System (NCMS) [40], and rural hypertensive patients could hardly get reimbursement for outpatient services. By contrast, urban employees covered by Urban Employee Basic Medical Insurance (UEBMI) could get adequate reimbursement for hypertension outpatient services [27, 41]. Less access to medical services and higher cost for outpatient services might be the main reasons that awareness, treatment, and control rates were higher among urban adults.

Nevertheless, the logistic analysis found that urban–rural disparities in hypertension awareness and treatment were not evident in 2015, and the GEE analysis showed these disparities narrowed significantly between 2 waves. In other words, awareness and treatment of rural adults increased faster than urban counterparts from 2011 to 2015, which indicated that urban–rural equity in hypertension management has been promoted. The primary health care system was getting stronger during this period and primary health care became more available and affordable in rural areas [21]. BNPHSP might make a great contribution because it serves to provide free blood pressure testing for permanent residents aged above 35 at their first visit to primary health care institutions [40]. People who were diagnosed with hypertension were directly included in hypertension management, and at least 4 face-to-face follow-up visits were required to provide for these patients per year. Studies demonstrated that BNPHSP had effectively improved the awareness, treatment, and control of hypertension [19, 42]. Rural residents were more likely to get BNPHSP and had higher effective follow-up rate compared with urban adults is a possible explanation of why these indicators increased faster among rural adults [39].

Although hypertension awareness, treatment, and control increased from 2011 to 2015, the overall rates of awareness, treatment, and control were still very low in both waves. Compared with developed countries, such as the United States, Korea, and Canada [43, 44], China still has a long way to enhance hypertension management. Efforts are still needed to be made to further improve hypertension management, especially in rural adults. Measures, such as strengthening the healthcare human resources, improving the quality of medical services, promoting public awareness programs, implementing health poverty alleviation programs, add access to affordable drugs, could be taken [8].

In accordance with some previous studies, BMI had a positive effect on hypertension awareness, treatment, and control [35, 45, 46]. Since BMI is a well-known risk factor for hypertension, overweight and obese adults might pay more attention to the management of hypertension [46]. Besides, current drinkers had less possibility of awareness, treatment, and control of hypertension. In addition, the richest adults had higher rates of hypertension awareness, treatment, and control than the poorest adults, which might be because richer people obtain more opportunities to be taught health-related knowledge [47], and poorer people have more difficulties to seek for medical services or persist taking medicine owing to unaffordable expenditure [48]. Free health examination and affordable hypertension management should be further provided to those poor residents.

The strengths of the present study included: First, the data we used is nationally representative and more up-to-date than most existing studies exploring urban–rural disparities in hypertension prevalence and management. Second, repeated cross-sectional survey designs were conducted in the present study using two waves of data to reveal the variation of urban–rural disparities in hypertension prevalence and management.

There are some limitations to this study. First, CHARLS is representative of Chinese adults ≥ 45 years rather than all Chinese adults. Second, excluding records with missing data on blood pressure tests may cause an overestimation of hypertension prevalence, for participants who refused to take body examination might be healthier than others. Third, a part of participants who refused to report household consumption were more likely to be urban rich residents, among which the awareness, treatment, and control rates were relatively high, so it could cause underestimation of urban–rural disparities in these indicators. Finally, only data from 2 waves had been used in this study, which might not fully reflect the trend of hypertension prevalence and management after the 2009 primary-care reform.

In conclusion, hypertension awareness, treatment, and control increased from 2011 to 2015, especially among rural adults, reflecting that the government of China had made an effort to achieve urban–rural equity in hypertension management. However, efforts are still needed to be made to enhance the management of hypertension, which was of a great distance from that of some developed countries. Besides, urban–rural inequity in hypertension management still exists. Residents with rural hukou, especially those with low economic status, should be given more care in hypertension management. Further policies, such as reforming the hukou system, making hypertension covered by all health insurance, and promoting blood pressure screening and health education programs in rural areas, could be implemented.

## Data Availability

The datasets generated and analyzed during the current study are publicly available in the CHARLS website [http://charls.pku.edu.cn].
